# A large-scale LC-MS dataset of murine liver proteome from time course of heavy water metabolic labeling

**DOI:** 10.1038/s41597-023-02537-w

**Published:** 2023-09-19

**Authors:** Henock M. Deberneh, Doaa R. Abdelrahman, Sunil K. Verma, Jennifer J. Linares, Andrew J. Murton, William K. Russell, Muge N. Kuyumcu-Martinez, Benjamin F. Miller, Rovshan G. Sadygov

**Affiliations:** 1 Department of Biochemistry and Molecular Biology, The University of Texas Medical Branch, Galveston, Texas USA; 2 Department of Surgery, The University of Texas Medical Branch, Galveston, Texas USA; 3 Sealy Center of Aging, The University of Texas Medical Branch, Galveston, Texas USA; 4 Department of Neuroscience, Cell Biology and Anatomy, The University of Texas Medical Branch, Galveston, Texas USA; 5 Aging and Metabolism Research Foundation, Oklahoma Medical Research Foundation, Oklahoma City, Oklahoma USA; 6 Oklahoma City VA, Oklahoma City, Oklahoma USA; 7 Present Address: Department of Molecular Physiology and Biological Physics, The University of Virginia, Charlottesville, Virginia USA

**Keywords:** Bioinformatics, Mass spectrometry, Proteomic analysis, Protein-protein interaction networks

## Abstract

Metabolic stable isotope labeling with heavy water followed by liquid chromatography coupled with mass spectrometry (LC-MS) is a powerful tool for *in vivo* protein turnover studies. Several algorithms and tools have been developed to determine the turnover rates of peptides and proteins from time-course stable isotope labeling experiments. The availability of benchmark mass spectrometry data is crucial to compare and validate the effectiveness of newly developed techniques and algorithms. In this work, we report a heavy water-labeled LC-MS dataset from the murine liver for protein turnover rate analysis. The dataset contains eighteen mass spectral data with their corresponding database search results from nine different labeling durations and quantification outputs from d2ome+ software. The dataset also contains eight mass spectral data from two-dimensional fractionation experiments on unlabeled samples.

## Background & Summary

Heavy water metabolic labeling followed by liquid chromatography coupled with mass spectrometry (LC-MS) is a powerful high throughput and large-scale technique for measuring the turnover rates of individual proteins *in vivo*^1–7^. In studies of mammalian species, an initial bolus of ^2^H_2_O is followed by deuterium-enriched drinking water (~8%) to rapidly equilibrate with the body water pool. This pool, in turn, rapidly labels proteogenic amino acids and generates ^2^H-labeled proteins^1^. All non-essential amino acids equilibrate to the enrichment of deuterium enrichment in the body water according to their numbers of non-labile hydrogens^8^. Labeling with heavy water is cost-effective^9^, easy to apply, and requires no dietary adaptation.

LC-MS profiles in deuterium labeled samples are mixtures of labeled and unlabeled forms of a peptide. The turnover rates of peptides/proteins can be determined from the time-course dynamics of the depletion of the relative abundance (RIA) of the monoisotope (M_0_)^10–12^. Bioinformatics tools, such as d2ome^12,13^, RIANA^3^, and DeuterRator^11^, automate protein turnover determination by calculating synthesis rates from mass shifts and predicted maximal peptide enrichment^14^. In the current work, we utilized d2ome+^15^ software to quantify the turnover rates of proteins and peptides from mass spectral data. This software quantified monoisotopic relative isotope abundances from partial isotope profiles. This technique minimizes the interferences from co-eluting contaminants in complex proteome mixtures and improves the accuracy of the estimation of label enrichment.

**Fig. 1:**
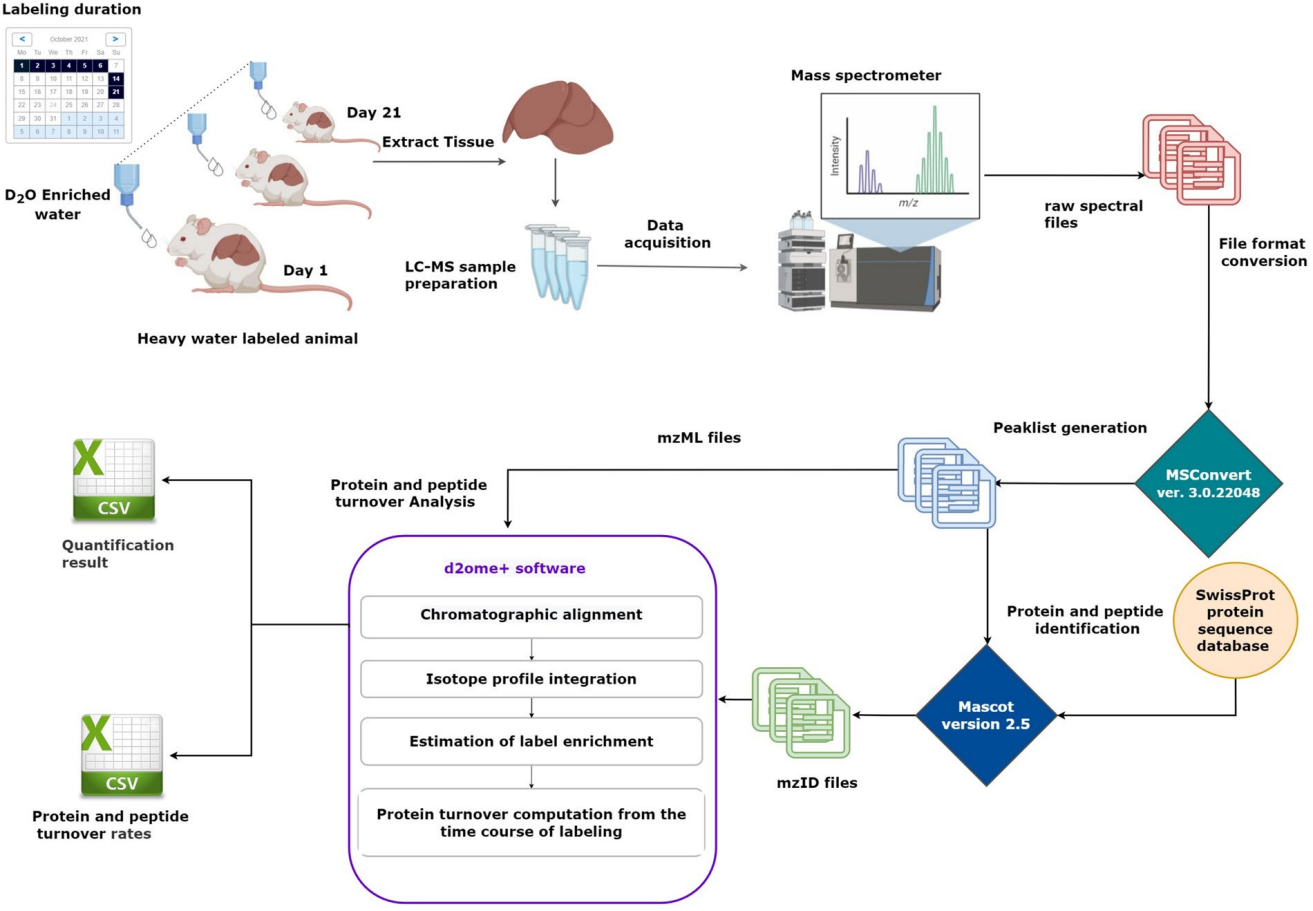
Workflow of the data collection and processing for proteome dynamics studies using stable isotope labeling and liquid-chromatography coupled to mass spectrometry. The figure depicts the main steps used in the experimental and data processing of protein turnover studies.

The dataset reported in this work was used in the development and validation of the d2ome+ algorithm. Figure 1 depicts the workflow used in the generation of the datasets reported in this data descriptor. As seen from the figure, the generation of datasets for protein turnover studies is laborious. It requires animal labeling, tissue harvesting at several durations of labeling, and the generation of LC-MS data of tissue proteome at each labeling duration.

Deuterium labeled samples analyzed with modern mass spectrometers are important for the development of algorithms for protein turnover analyses. A few large-scale and high-throughput LC-MS datasets of heavy water metabolically labeled samples have been published^3,10–12^. Lau and colleauges^10^ provided datasets of cardiac samples from six mouse strains^10^. Subcellular fractionation provided proteomic data sets from different cellular compartments (mitochondria, nuclei, and cytoplasmic), which were analyzed using LTQ Orbitrap Elite mass spectrometer, coupled on-line to the nano-UPLC system through a Thermo EasySpray interface. We published^12^ large-scale mass spectral data of heavy water labeled mouse model of Fatty Liver Diseases, LDLR^−/−^. The murine liver proteome was fractionated by SDS-PAGE using AnyKD gel. The proteomes were analyzed by Ultimate 3000 UHPLC (Thermo Scientific, CA) coupled on-line to Q Exactive™ Plus Hybrid Quadrupole-Orbitrap™ Mass Spectrometer. Hammond and colleagues^3^ reported large-scale LC-MS datasets from four different mouse tissues (liver, kidney, heart, and muscle). The labeling duration was densely sampled at the early labeling durations. The chosen tissues provide samples for fast (liver and kidney) and slow (heart and muscle) proteome dynamics. The turnover rates obtained from heavy water labeling can be compared with those obtained from heavy Lys labeling, which they also provided. The proteomes were analyzed using a Q-Exactive HF quadrupole-Orbitrap mass spectrometer coupled to a Dionex Ultimate 3000 RSLC nano-liquid chromatograph. Naylor and colleagues^11^ reported LC-MS data of mouse blood serum samples labeled with heavy water. The mass spectral datasets were collected using an Agilent 6530B QToF, a Thermo Orbitrap XL, and a Thermo Fusion Tribrid. Different settings for mass resolution were applied in Thermo Fusion Tribird: at 30 K, 60 K, 120 K, 240 K, and 480 K mass resolutions. These datasets provide opportunities to determine the efficiency and accuracy of turnover rate estimations at various resolutions. In general, at very high resolutions, the number of identified peptides and the accuracy of turnover rate estimations decreased^16^.

The fidelity of protein turnover rate determination depends on a number of parameters used in the modeling. It includes the mass accuracy and resolution of the mass analyzer used to generate spectral data^16^, the accuracy of the number of exchangeable hydrogens^17^, the estimation of label enrichment from the composite isotope profiles^18^, and the spacing of sampling time points^19^. Generally, there is a need for datasets from different model organisms (including different strains of the same species), studied under different biological conditions (e.g., age, treatment status), and obtained using different LC-MS instrumentation and settings. Many technical issues, such as the type of mass analyzers, chromatographic separation techniques, and database search engines, can influence the outcome of data analyses. For the same species, some of the many factors that regulate protein turnover are tissue type and age. It is also affected by protein localization, ribosome availability, aggregation, post-translational modification, cellular energetics, and redox state, among others.

The current work describes our dataset used in the proteome dynamics analyses of murine liver, recently published in Communications Chemistry^15^. The raw mass spectral data was acquired from eighteen murine liver tissue using a Thermo Orbitrap Eclipse mass spectrometer. The raw mass spectral data were converted to mzML format using proteowizard MSConvert tool^20^ version 3.0.22048. The Mascot database search engine^21^ (version 2.5) and SwissProt protein sequence database (12 February 2022 release) were used to identify peptides from tandem mass spectra data. Both the spectral data (in mzML format) and the database search results (in mzID format) were used as input for d2ome+ software for quantifying the turnover rates of proteins and peptides. The differences from the previously reported datasets include a large number of peptide spectrum matches (PSMs), dense sampling of labeling duration, long chromatographic times, and retention time alignment. The data analysis results include quantification of label enrichment for peptides in experiments in which they were not identified via MS/MS (by retention time alignment and transfer of peptide identifications).

d2ome+ output includes peak detection and quantification for every identified peptide of every protein (Protein_Name.Quant.csv) and their corresponding turnover rates (Protein_Name.RateConst.csv). The Protein_Name.Quant.csv file contains comprehensive information about each peptide of a protein. For each peptide, the file contains its amino acid sequence, charge state, theoretical m/z, theoretical isotope abundances^22,23^, precursor m/z, the highest Mascot Ion score, Mascot expectation, mass accuracy (in ppm), scan number, the integrated abundances of six mass isotopomers from MS1 scans, chromatographic elution start and end times^12,24^. The Protein_Name.RateConst.csv file contains the peptides sequence, its rate constant and corresponding confidence intervals, and other statistical information about the computed rates^12,24^.

## Methods

### Experimental procedures

#### Animal labeling experiments

Eighteen four-month-old *C57/BL6J* male mice were used in the time course experiment. Two randomly selected mice were not labeled, and their tissues were used to estimate natural isotope abundances. The remaining sixteen mice were IP injected with 750–960 ul of 99.9% D_2_O made isotonic with 0.9 g NaCl w/v^25^. Immediately afterward, mice were given free access to 8% enriched (v/v) deuterated water for 1, 2, 3, 4, 5, 6, 14, and 21 days of labeling durations. At each time point, two mice were euthanized with CO_2_, and dissected livers were flash-frozen in liquid nitrogen and stored at −80 °C. The body water enrichment in deuterium was determined using a blood sample collected from mice via cardiac puncture using a syringe with a 25 G needle and the procedure described in a previous study^26^. Mouse experiments were carried out according to the Guide for Care and Use of Laboratory Animals by the National Research Council and approved by the Institutional Animal Care and Use Committee of UTMB. A UTMB veterinarian technician performed IP injection, euthanization, and tissue extractions.

#### Sample preparation

100 mg of the murine liver was diced and placed into 0.5 mL of 5% SDS, 50 mM TEAB, 1 mM TCEP solution in a clean 2 mL Nunc screw cap tube. 3uL of benzonase nuclease (Sigma) was added to the solution, followed by 0.5 mL of 1.0 mm zirconia/silica beads (BioSpec) homogenized by bead beating, which was performed for 20 seconds using a Soni-Beast (BioSpec). The sample was subjected to a second round of bead beating and repeated as necessary to achieve the full homogenization of the tissue. The sample was cooled to room temperature, and 2 μL of 0.5 M iodoacetamide acid was added and allowed to react for 20 minutes in the dark. The supernatant was transferred to a clean 15 mL Falcon tube. 2 μL of freshly prepared 0.5 M Dithiothreitol was added to the sample, and it was incubated at room temperature for ten minutes. Proteins were precipitated using 3 mL of ice-cold acetone and centrifuged after storing overnight at −20 °C. Acetone was decanted, and the resulting pellet diluted in 5% SDS, 50 mM TEAB, pH 7.1, to a final volume of 200 μL. The samples were then centrifuged at 17,000 g for 10 minutes to remove any debris. Next, 2.75 μL of 12% phosphoric acid was added to the protein solution, and 165 μL of binding buffer (90% methanol, 100 mM TEAB, final pH 7.1) was then added to the solution. The resulting solution was added to S-Trap spin column (protifi.com) and passed through the column using a benchtop centrifuge (30-second spin at 4,000 g). The spin column was washed with 400 μL of binding buffer (90% methanol, 100 mM TEAB, pH = 7.55) and centrifuged. This was repeated two more times. Trypsin was added to the protein mixture in a ratio of 1:25 in 50 mM TEAB, pH 8, and incubated at 37 °C for four hours. Peptides were eluted with 80 μL of 50 mM TEAB, followed by 80 μL of 0.2% formic acid, and finally 80 μL of 50% acetonitrile and 0.2% formic acid. The combined peptide solution was then dried in a speed vac and resuspended in 2% acetonitrile, 0.1% formic acid, and 97.9% water and placed in an autosampler vial.

#### LC-MS measurements

Nano-flow LC-ESI-MS measurements were performed using a Dionex Ultimate 3000 UHPLC system coupled online to an Eclipse Orbitrap mass spectrometer (Thermo Fisher Scientific, San Jose, CA) through a nanospray ion source. Peptides were delivered directly to the analytical column (75 µm × 25 cm, packed with 1.6 µm C18 resin, Aurora, IonOpticks). After equilibrating the column in 99% solvent A (0.1% formic acid in water) and 1% solvent B (0.1% formic acid in acetonitrile (ACN)), the samples (2 µL in solvent A), the sample was eluted with a flow rate of 400 nl/min as follows: isocratic at 2% B, 0–5 min; 2% to 5% 5–6 min, 4% to 25% B, 6–170 min; 25% to 38% B, 170–196 min; 38% to 90% B, 196–199 min; isocratic at 90% B, 199–200 min; 90% to 5%, 200–201 min; 5% 201–202.5 min; 5%-90% 202.5–203 min, isocratic at 90% B, for 1 min and 90% to 1%, 204–205 min and held there for 15 minutes. The Eclipse Orbitrap was operated in data-dependent acquisition (DDA) and positive ionization mode. Full scan MS1 spectra were recorded in the Orbitrap from 380 to 1400 m/z at a resolution of 60,000 (at an m/z = 200, automatic gain control (AGC) target value of 4e5 ions, and a maximum injection time (maxIT) of 50 ms). MS/MS spectra were recorded in the ion trap mass analyzer after HCD (higher energy collisional dissociation) fragmentation using isolation window 1.4 m/z, AGC target value of 1e4, maxIT of 35 ms, 30% normalized collision energy (NCE), and dynamic exclusion time was 30 s. Precursor ions with charges of +2 to +8 were selected for MS/MS sequencing.

#### Fractionation of peptides of the unlabeled samples

Proteolytic digests from the (unlabeled mouse) murine liver proteins were loaded onto an equilibrated, high-pH, reversed-phase fractionation spin column (Thermo Cat#84868). Peptides bound to the resin under aqueous conditions were desalted by washing the column with water using low-speed centrifugation. The bound peptides were eluted into eight different fractions by application of a step gradient of increasing acetonitrile concentrations (5–50% Acetonitrile, each with 0.1% Triethylamine). They were collected using centrifugation. Each of the fractions was dried in a vacuum centrifuge and reconstituted in 2% acetonitrile, 1% acetic acid, and 0.08% formic acid. They were put in an autosampler for a 90-minute gradient LC-MS analysis. The same mass spectrometer settings as for the non-fractionated samples were applied.

#### Measurements of body water enrichment in deuterium

Isotope ratio mass spectrometry (IRMS) was used to measure body water deuterium enrichment in plasma, as described previously^26^. 10 µl of plasma was diluted to a volume of 200 µl in water and placed in an Exetainer® (Labco Ltd., UK), along with 5 mg of activated charcoal, 200 mg of copper powder, and a platinum catalytic rod. Afterward, the Exetainer® was loaded onto a Gasbench II online gas preparation and introduction system (ThermoFisher Scientific, MA, USA), where the sample was flushed for 7 min with 2% H_2_ in helium. The sample was allowed to equilibrate for 4 h. On equilibration, an aliquot of the gas headspace was injected into a Delta V Advantage isotope ratio mass spectrometer (ThermoFisher Scientific, MA, USA). The ratio of deuterium to hydrogen was determined in duplicate. The linearity of the machine over the expected range of deuterium body water enrichment was verified by the generation of a standard curve using 99.9% deuterium-enriched water (Sigma-Aldrich, St. Louis, MO, USA).

## Data Records

All raw mass spectral data, Mascot search results, and d2ome+ quantification results have been deposited in the MassIVE^27^ (http://massive.ucsd.edu) repository with the identifier MSV000090148^28^.

### Data record 1

Mass spectrometric data acquired using Thermo Orbitrap Eclipse mass spectrometer from *C57/BL6J* male mice liver is reported in this work. The dataset contains eighteen raw mass spectral data, their corresponding mzML files converted using proteowizard MSConvert tool^20^ version 3.0.22048 and Mascot^21^ database search engine (version 2.5) results in mzID format. In addition, the dataset contains the configuration file and quantification result from the d2ome+ software. The output of the software includes peak detection and quantification for every peptide of every protein (Protein_Name.Quant.csv) and their corresponding rate constants (Protein_Name.RateConst.csv. Table 1 presents the spectral file name, database search result, body weight enrichment (BWE), and labeling duration information for each biological replicate experiment.

**Table 1:** Mass spectral data, database search results, labeling duration, and body water enrichment of biological replicates.

Mass spectral file name	Database search result	Body water enrichment	Labeling duration
220min_OT60it_26Aug21_RSL_01.mzML	220min_OT60it_26Aug21_RSL_01.mzid	0	0
DO2_mouseliver_wr_28feb2022_274.mzML	DO2_mouseliver_wr_28feb2022_274.mzid	0	0
220min_OT60it_26Aug21_RSL_05.mzML	220min_OT60it_26Aug21_RSL_05.mzid	0.0304	1
220min_OT60it_26Aug21_RSL_14.mzML	220min_OT60it_26Aug21_RSL_14.mzid	0.0235	1
220min_OT60it_26Aug21_RSL_13.mzML	220min_OT60it_26Aug21_RSL_13.mzid	0.0322	2
220min_OT60it_26Aug21_RSL_19.mzML	220min_OT60it_26Aug21_RSL_19.mzid	0.0325	2
220min_OT60it_26Aug21_RSL_08.mzML	220min_OT60it_26Aug21_RSL_08.mzid	0.0281	3
220min_OT60it_26Aug21_RSL_18.mzML	220min_OT60it_26Aug21_RSL_18.mzid	0.0309	3
220min_OT60it_26Aug21_RSL_04.mzML	220min_OT60it_26Aug21_RSL_04.mzid	0.0257	4
220min_OT60it_26Aug21_RSL_06.mzML	220min_OT60it_26Aug21_RSL_06.mzid	0.0259	4
220min_OT60it_26Aug21_RSL_02.mzML	220min_OT60it_26Aug21_RSL_02.mzid	0.0359	5
220min_OT60it_26Aug21_RSL_07.mzML	220min_OT60it_26Aug21_RSL_07.mzid	0.0287	5
220min_OT60it_26Aug21_RSL_12.mzML	220min_OT60it_26Aug21_RSL_12.mzid	0.0265	6
220min_OT60it_26Aug21_RSL_16.mzML	220min_OT60it_26Aug21_RSL_16.mzid	0.0359	6
220min_OT60it_16Sept21_RSL_20.mzML	220min_OT60it_16Sept21_RSL_20.mzid	0.0485	14
220min_OT60it_26Aug21_RSL_09.mzML	220min_OT60it_26Aug21_RSL_09.mzid	0.0473	14
220min_OT60it_26Aug21_RSL_15.mzML	220min_OT60it_26Aug21_RSL_15.mzid	0.0374	21
220min_OT60it_26Aug21_RSL_17.mzML	220min_OT60it_26Aug21_RSL_17.mzid	0.0399	21

The data deposited to MassIVE contains different directories, including raw, peak, quant, and result. The raw spectral data is stored under the folder name raw. The mzML formatted spectral data is placed under the folder peak. The Mascot database search result and quantification output from d2ome+ software are stored in the results and quant folder, respectively. The quant folder also contains both the parameter (files.txt) and configuration (quant.state) files to re-run d2ome+ software.

In addition to the quantification results, this dataset contains retention time (RT) alignment information from pairs of raw mass spectral data. Retention time alignment has been widely used to address missing value problems in the data acquired using data-dependent accusation mode^29^. In this study, we utilized correlation-optimized warping of raw mass spectral data technique to generate the RT alignment of LC-MS-MS/MS experiments^30,31^. The reported dataset contains 17 RT alignment “.csv” files that are generated sequentially between two successive (in the labeling duration) experiments. Each “.csv” file contains a list of retention times from the reference and the corresponding sample experiment. The files can be accessed from the MassIVE repository^28^ using the following link, ftp://massive.ucsd.edu/MSV000090148/updates/2023-06-02_henockmamo54_92192285/quant/chromatographic_alignment/. Table 2 (up to four days of labeling) and 3 (from four to up 21 days of labeling) present the name of the reference experiment, sample experiment, and resulting RT alignment information “.csv” file. The software used to generate the chromatographic RT alignment is available on GitHub (https://github.com/rgsadygov/ChromatographicAlignment/releases/tag/v1.0.0).

**Table 2:** Retention time alignment of LC-MS-MS/MS experiments of samples up to four days of labeling with heavy water.

Reference experiment	Sample experiment	Retention time alignment
220min_OT60it_26Aug21_RSL_01.mzML	DO2_mouseliver_wr_28feb2022_274.mzML	Alignment_of_220min_OT60it_26Aug21_RSL_01_DO2_mouseliver_wr_28feb2022_274.csv
DO2_mouseliver_wr_28feb2022_274.mzML	220min_OT60it_26Aug21_RSL_05.mzML	Alignment_of_DO2_mouseliver_wr_28feb2022_274_220min_OT60it_26Aug21_RSL_05.csv
220min_OT60it_26Aug21_RSL_05.mzML	220min_OT60it_26Aug21_RSL_14.mzML	Alignment_of_220min_OT60it_26Aug21_RSL_05_220min_OT60it_26Aug21_RSL_14.csv
220min_OT60it_26Aug21_RSL_14.mzML	220min_OT60it_26Aug21_RSL_13.mzML	Alignment_of_220min_OT60it_26Aug21_RSL_14_220min_OT60it_26Aug21_RSL_13.csv
220min_OT60it_26Aug21_RSL_13.mzML	220min_OT60it_26Aug21_RSL_19.mzML	Alignment_of_220min_OT60it_26Aug21_RSL_13_220min_OT60it_26Aug21_RSL_19.csv
220min_OT60it_26Aug21_RSL_19.mzML	220min_OT60it_26Aug21_RSL_08.mzML	Alignment_of_220min_OT60it_26Aug21_RSL_19_220min_OT60it_26Aug21_RSL_08.csv
220min_OT60it_26Aug21_RSL_08.mzML	220min_OT60it_26Aug21_RSL_18.mzML	Alignment_of_220min_OT60it_26Aug21_RSL_08_220min_OT60it_26Aug21_RSL_18.csv
220min_OT60it_26Aug21_RSL_18.mzML	220min_OT60it_26Aug21_RSL_04.mzML	Alignment_of_220min_OT60it_26Aug21_RSL_18_220min_OT60it_26Aug21_RSL_04.csv
220min_OT60it_26Aug21_RSL_04.mzML	220min_OT60it_26Aug21_RSL_06.mzML	Alignment_of_220min_OT60it_26Aug21_RSL_04_220min_OT60it_26Aug21_RSL_06.csv

**Table 3:** Retention time alignment of LC-MS-MS/MS experiments of samples from four up to 21 days of labeling with heavy water.

Reference experiment	Sample experiment	Retention time alignment
220min_OT60it_26Aug21_RSL_06.mzML	220min_OT60it_26Aug21_RSL_02.mzML	Alignment_of_220min_OT60it_26Aug21_RSL_06_220min_OT60it_26Aug21_RSL_02.csv
220min_OT60it_26Aug21_RSL_02.mzML	220min_OT60it_26Aug21_RSL_07.mzML	Alignment_of_220min_OT60it_26Aug21_RSL_02_220min_OT60it_26Aug21_RSL_07.csv
220min_OT60it_26Aug21_RSL_07.mzML	220min_OT60it_26Aug21_RSL_12.mzML	Alignment_of_220min_OT60it_26Aug21_RSL_07_220min_OT60it_26Aug21_RSL_12.csv
220min_OT60it_26Aug21_RSL_12.mzML	220min_OT60it_26Aug21_RSL_16.mzML	Alignment_of_220min_OT60it_26Aug21_RSL_12_220min_OT60it_26Aug21_RSL_16.csv
220min_OT60it_26Aug21_RSL_16.mzML	220min_OT60it_16Sept21_RSL_20.mzML	Alignment_of_220min_OT60it_26Aug21_RSL_16_220min_OT60it_16Sept21_RSL_20.csv
220min_OT60it_16Sept21_RSL_20.mzML	220min_OT60it_26Aug21_RSL_09.mzML	Alignment_of_220min_OT60it_16Sept21_RSL_20_220min_OT60it_26Aug21_RSL_09.csv
220min_OT60it_26Aug21_RSL_09.mzML	220min_OT60it_26Aug21_RSL_15.mzML	Alignment_of_220min_OT60it_26Aug21_RSL_09_220min_OT60it_26Aug21_RSL_15.csv
220min_OT60it_26Aug21_RSL_15.mzML	220min_OT60it_26Aug21_RSL_17.mzML	Alignment_of_220min_OT60it_26Aug21_RSL_15_220min_OT60it_26Aug21_RSL_17.csv

### Data record 2

Raw mass spectral data and the corresponding database search results of murine liver unlabeled samples from two-dimensional fractionation experiments are incorporated into the dataset. The fractionated samples were separated by high pH reverse phase. Table 4 shows each pair of the fractionated dataset and their organic solvent concentration. The accession number for the MassIVE repository is MSV000090148^28^ and can be accessed using the following link, ftp://massive.ucsd.edu/MSV000090148/updates/2023-03-21_henockmamo54_a873f4ee/

**Table 4:** Mass spectral data of the fractionated peptides from the unlabeled samples.

Experiment name	Organic sovent concentration
do-2-5pct_90mingrad_60OTit_1p3s_cycletime_E_wr_03Feb23_006	5.0% acetonitrile in triethylamine
do-2-7p5pct_90mingrad_60OTit_1p3s_cycletime_E_wr_03Feb23_007	7.5% acetonitrile in triethylamine
do-2-10pct_90mingrad_60OTit_1p3s_cycletime_E_wr_03Feb23_008	10% acetonitrile in triethylamine
do-2-12p5pct_90mingrad_60OTit_1p3s_cycletime_E_wr_03Feb23_009	12.5% acetonitrile in triethylamine
do-2-15pct_90mingrad_60OTit_1p3s_cycletime_E_wr_03Feb23_010	15% acetonitrile in triethylamine
do-2-17p5pct_90mingrad_60OTit_1p3s_cycletime_E_wr_03Feb23_011	17.5% acetonitrile in triethylamin
do-2-20pct_90mingrad_60OTit_1p3s_cycletime_E_wr_03Feb23_012	20% acetonitrile in triethylamine
do-2-50pct_90mingrad_60OTit_1p3s_cycletime_E_wr_03Feb23_013	50% acetonitrile in triethylamine

### Data record 3

Raw mass spectral data files from IRMS of murine blood samples (36 dxf format files) and from three standard runs (21 dxf files), extracted isotope distributions (Raw Data.xls), and the corresponding processed data (IRMS Standard Curves and Results.xls) used for the measurements of body water enrichment comprised the Data record 3. The instrument files contain replicate measurements of isotope distributions of hydrogens for each biological replicate at every time point of labeling (e.g., Day4_BiolRep1_TechnicalRep1.dxf for the first technical replicate of the first biological replicate labeled with heavy water for four days). For each individual run, a standard containing ^2^H_2_O at varying concentrations was prepared, with concentrations reported in parts per million (ppm) (e.g., Run1_Std_10ppm.dxf). Isobaric interference from ^1^H_3_^+^ formation by the ion source was also determined at the start of each run (e.g., Run1_H3factor.dxf for the first run). Sample deuterium enrichment was calculated according to the standard curve produced for each run (IRMS Standard Curves and Results.xls).

The accession number of this data record in the MassIVE repository is MSV000090148^28^. It can be accessed using the following link, ftp://massive.ucsd.edu/MSV000090148/updates/2023-07-18_henockmamo54_e3d58a4b/other/IRMS/.

## Technical Validation

**Fig. 2:**
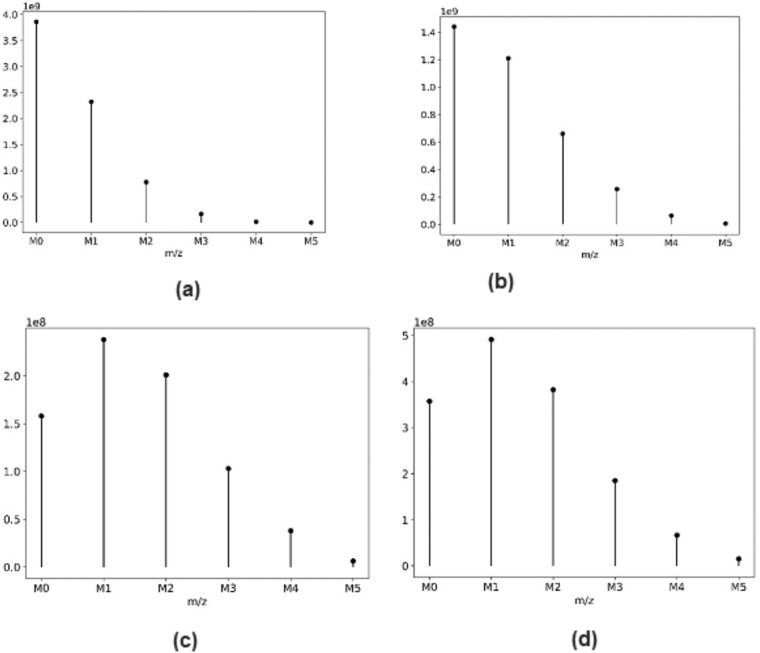
The monoisotopic RIA depletes with the labeling duration. Isotope profiles of KQTALAELVK^+2^ peptide (**a**) from an unlabeled sample (**b**) from a labeled sample (day 3) (**c**) from a labeled sample (day 14) (**d**) from a labeled sample (day 21).

**Fig. 3:**
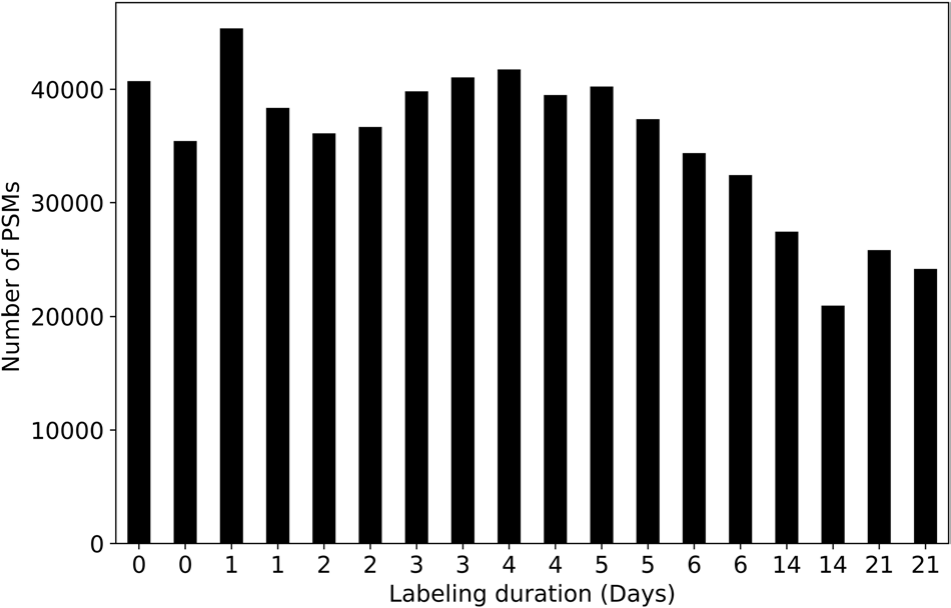
The number of peptide spectrum matches (PSMs) that passed the 1% false discovery rate (FDR) threshold. The number of PSMs decreases with the increased labeling duration.

In heavy water metabolic labeling, the number of identified peptide spectrum matches (PSMs) decreases with increased labeling duration. The factors that affect the peptide identification and quantification in heavy water labeling experiments are the stochastic nature of the data-dependent acquisition (DDA) and the selection of non-monoisotopic mass isotopomers for peptide fragmentation^32^. In addition, the changes in the isotope profiles due to the incorporation of deuterium into non-essential amino acids (NEAAs) reduce the number of identified PSMs by the conventional database search technique^33,34^. The spectral profile of each peptide is unique for the type of animal, tissue, and labeling duration. Conventional database search techniques, which identifies peptides from tandem mass spectra and protein sequence databases, show performance deterioration as the labeling duration increase and the isotope profile of the peptides changes. The reported dataset presented the quantification results for each identified peptide for each labeling duration. Figure 2 depicts the isotope profile of the KQTALAELVK^+2^ peptide of ALBU_MOUSE protein at four different labeling durations. Figure 3 shows PSMs that passed the 1% false discovery rate (FDR) threshold at nine different time points of labeling and eighteen experiments from the murine liver.

**Fig. 4:**
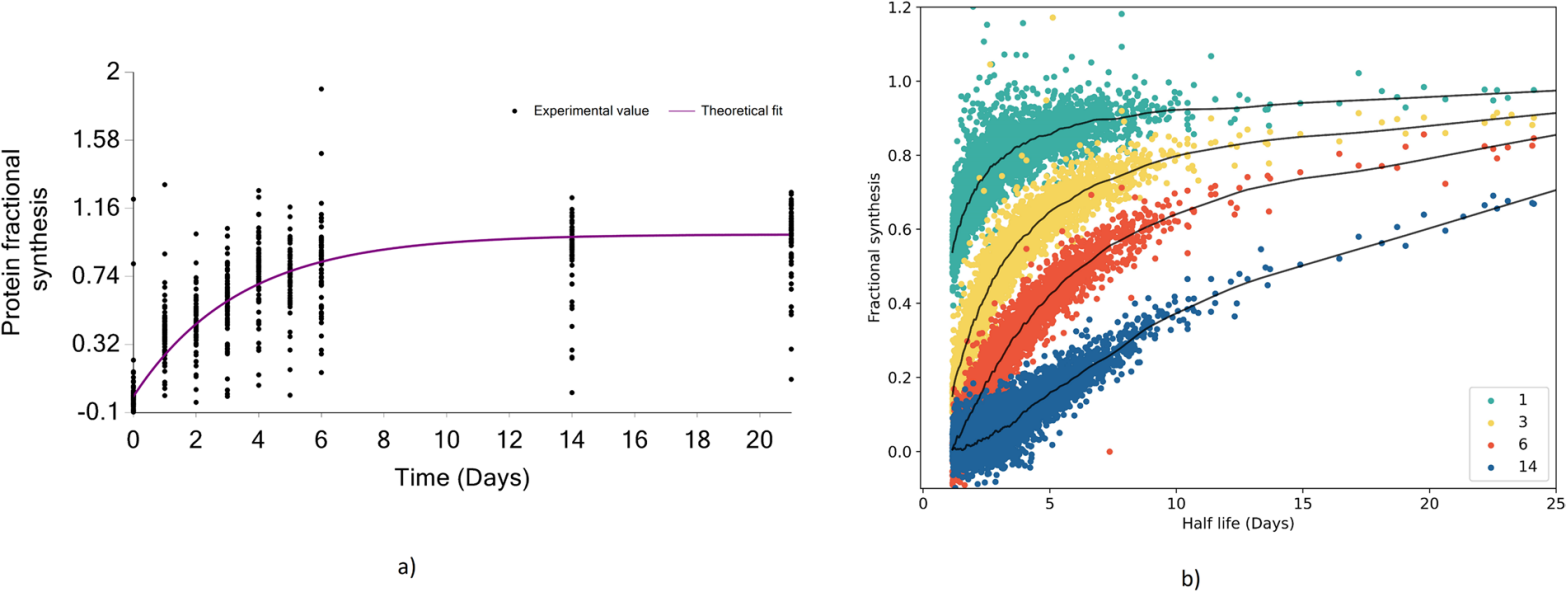
Turnover rate analysis for murine liver protein. (**a**) The fractional synthesis rate of ALBU_MOUSE, the scatter plot indicates the fractional value of peptides in the ALBU_MOUSE protein. The solid line shows the fit from the computed turnover rate for the protein (**b**) Fractional synthesis of all liver peptides at four different labeling durations – 1, 3, 6, and 14 days. Shown in black are the LOESS fits to the data from each labeling duration.

Database search and identification for proteins and peptides were conducted using the Mascot search engine. Each peptide spectrum match (PSM) was compared based on ion score significance thresholds, and peptides with high ion scores and expectations were considered significant peptides. To ensure the quality of the reported output, all PSMs were filtered to meet <1% false discovery rate (FDR). Figure 4 depicts the fractional synthesis rate (FSR) and time course quantification results along with labeling duration (x-axis) for the ALBU_MOUSE protein. The fractional synthesis rate^9^, the relative proportion of the labeled peptides normalized to the asymptotic labeling, serves as a quantitative measure of the labeling. It is noted that in the case of incomplete labeling (the body water enrichment in deuterium did not exceed 5% in any of the experimental subjects in this study), the newly synthesized proteins contain labeled and unlabeled forms. The asymptotic labeling reaches equilibrium with the precursor enrichment. The graph presents the experimental FSR of each peptide of the protein as a scatter plot and the theoretical fit computed based on the protein rate constant as a solid line.

### Experimental contaminants as positive controls for zero turnover rate computation

**Fig. 5:**
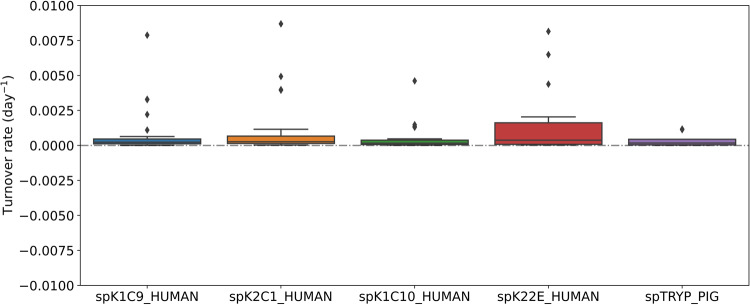
Fractional synthesis rate for the common contaminant proteins. Shown are the protein turnover rates (day^−1^) for several commonly known contaminant proteins, spK1C9_HUMAN, spK2C1_HUMAN, spK1C10_HUMAN, spK22E_HUMAN, and spTRYP_PIG.

We note that in addition to the murine liver proteins, the sample also contained well-known contaminants such as trypsin and various human keratins. These proteins are contaminants that result during sample preparation^35^. Their sequences were added to the protein sequence database used by Mascot. The contaminants are not labeled with heavy water; therefore, they serve as controls for a zero turnover rate. Figure 5 shows the results of quantifications of several of these proteins from a large number of peptides. The time courses of the monoisotopic RIAs are flat for the peptides of these proteins. The computed rates of proteins were practically zero.

### Mascot score and expectation values

**Fig. 6:**
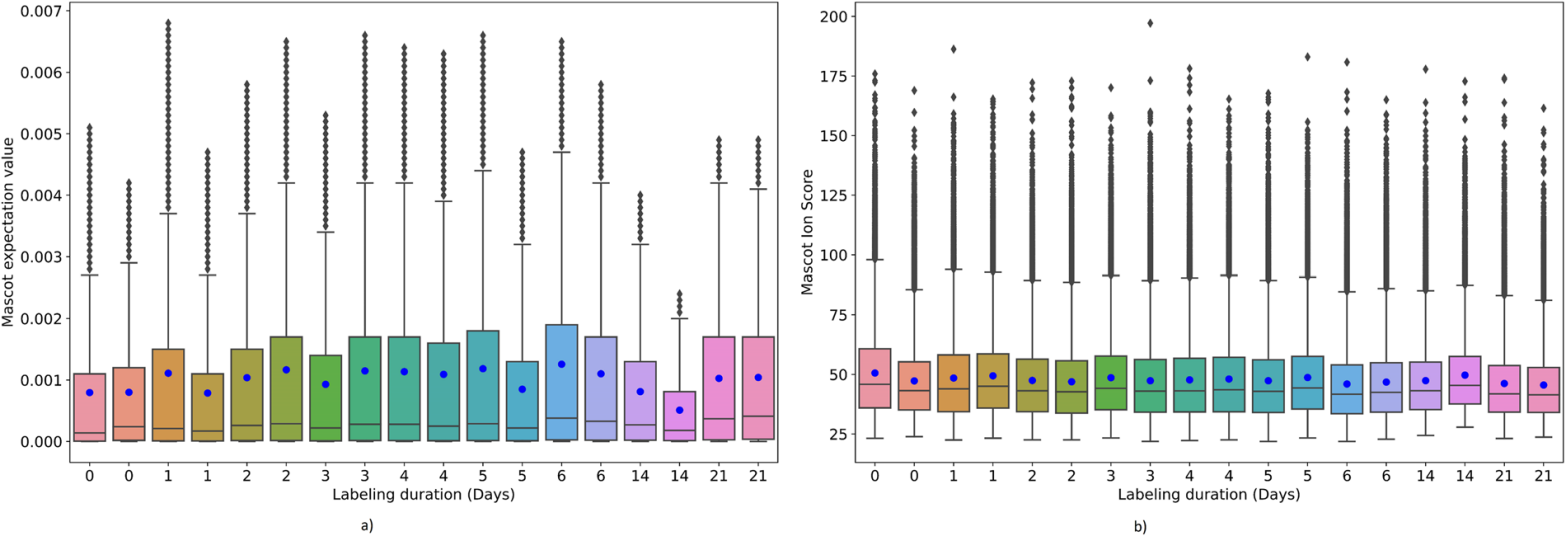
Mascot database search result validation metrics distribution. (**a**) peptide spectrum matches expectation values for different labeling durations (**b**) distribution of ion score values for multiple labeling durations.

the database search result contains multiple quality metrics for peptide spectrum matches, including expectation and peptide ion score values. Expectation metrics indicate the number of times the spectrum match may occur by random. The box plot in Fig. 6a,b depicts the distribution of expectation values and peptide scores in the eighteen experiments, respectively. The blue dot and the vertical bar inside the box indicate the mean and median values of the distribution. The top and bottom edges of the box indicate the 1^st^ and 3^rd^ quartiles of the distribution. Overall, the mean, median, and standard deviation of the expectation values for PSMs are 0.001, 0.00025, and 0.0014, respectively. Similarly, the mean, median, and standard deviation values for PSMs ion score are 47.83, 43.42, and 17.78, respectively.

#### The goodness of fit characteristics of quantified peptides

**Fig. 7:**
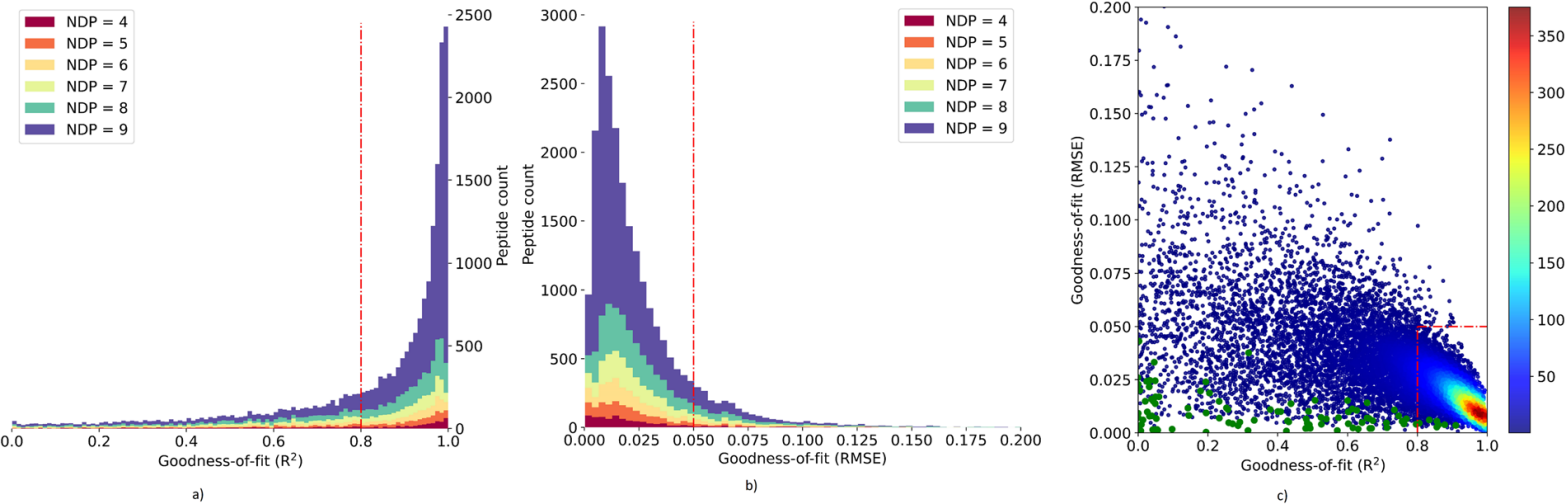
The distributions of GOF characteristics, (**a**) R^2^ of the experimental time course and theoretical fit; (**b**) root mean squared error(RMSE) as a function of the number of data points (various labeling durations); (**c**) the scatter plot of the GOF characteristics of a peptide, R^2^ (x-axis) and RMSE (y-axis). The green dots indicate slow-turning peptides with acceptable GOF characteristics (RMSE ≤ 0.05), and the red dotted lines indicate the cutoff threshold for GOF characteristics. The red box in the bottom right corner of the graph shows the acceptable regions of the distribution and accounts for 63% of all peptides.

In heavy water metabolic labeling, the gradual incorporation of deuterium into peptides reduces the monoisotopic RIA. The monoisotopic RIA can be computed either from the complete or partial isotope profiles. The quantification results reported in this work have utilized the partial as well as complete isotope profiles. This approach is reported in a recent study^13^, which demonstrated that the usage of partial isotope profiles increased the proteome coverage and alleviated interferences caused by co-eluting contaminants. In the course of the development of the approach, this dataset was thoroughly examined and validated. Several metrics, including the coefficient of determination (R^2^), root mean squared error (RMSE), and the Pearson correlation coefficient, were used to determine the goodness of fit (GOF) between the quantified experimental data and computed theoretical values. To determine the acceptable GOF ranges for peptides, we used R^2^ ≥ 0.8 and RMSE ≤ 0.05 cutoff as thresholds. For peptides with relatively slow turnover rates (k ≤ 0.01 day^−1^), we used RMSE ≤ 0.05 as a GOF threshold. In peptides that were quantified in multiple labeling durations, a higher proportion was closely fitted to the theoretical values and passed the designated GOF thresholds (73% of peptides quantified in nine time points; 56% of peptides quantified in eight time points; 50% of peptides quantified in seven time points; 47% of peptides quantified in six time points; 49% of peptides quantified in five time points; 48% of peptides quantified in four time points;). Overall, 62% of peptides with four or more data points (various labeling durations) passed the GOF measures of thresholds. Compared to a previous study^9,13^ that reported 35–45% of high GOF and usable quantified peptides, this dataset reported relatively increased proteome coverage and a high proportion of peptides that are useful for protein turnover rate estimation. Figure 7a,b depict the distributions of the numbers of PSMs (y-axis) as functions of GOF characteristics for various NDP values (x-axis). Figure 7c shows the scatter plot of RMSE and R^2^ values of the quantified peptides. The red box in the bottom right corner indicates the acceptable GOF range, and the green dots indicate peptides with slow turnover rates and RMSE ≤ 0.5.

## Data Availability

The executables and instructions for d2ome+ software are available on GitHub, https://github.com/rgsadygov/d2ome/releases/tag/v1.05. The retention time alignment software and its instructions are available on GitHub, https://github.com/rgsadygov/ChromatographicAlignment/releases/tag/v1.0.0.
